# Massive Fetomaternal Hemorrhage Caused by an Intraplacental Choriocarcinoma: Case Report and Review of the Literature

**DOI:** 10.3390/diagnostics14212454

**Published:** 2024-11-01

**Authors:** Felice Sorrentino, Teresa Silvestris, Francesca Greco, Lorenzo Vasciaveo, Guglielmo Stabile, Veronica Falcone, Andrea Etrusco, Antonio D’Amato, Antonio Simone Laganà, Luigi Nappi

**Affiliations:** 1 Department of Medical and Surgical Sciences, Institute of Obstetrics and Gynecology, University of Foggia, 71121 Foggia, Italy; teresa.silvestris@unifg.it (T.S.); grecofrancesca1989@gmail.com (F.G.); lvasciaveo@gmail.com (L.V.); guglielmost@gmail.com (G.S.); luigi.nappi@unifg.it (L.N.); 2 Pathology Unit, PoliclinicoRiuniti, University of Foggia, 71122 Foggia, Italy; vero.fal90@gmail.com; 3 Unit of Obstetrics and Gynecology, “Paolo Giaccone” Hospital, Department of Health Promotion, Mother and Child Care, Internal Medicine and Medical Specialties (PROMISE), University of Palermo, 90127 Palermo, Italy; etruscoandrea@gmail.com (A.E.); antoniosimone.lagana@unipa.it (A.S.L.); 4 Department of Interdisciplinary Medicine (DIM), Unit of Obstetrics and Gynecology, University of Bari “Aldo Moro”, Policlinico of Bari, Piazza Giulio Cesare 11, 70124 Bari, Italy; antoniodamato19@libero.it

**Keywords:** intraplacental choriocarcinoma (IC), massive fetomaternal hemorrhage (FMH), placenta, gestational trophoblastic disease

## Abstract

Intraplacental choriocarcinoma (IC) is a gestational trophoblastic neoplasia located within the placenta. Due to its silent presentation, more than half of the cases are diagnosed incidentally. An association with fetomaternal hemorrhage (FMH), stillbirth, and intrauterine growth restriction has been found. The aim of this review is to describe the clinical management of this rare condition stemming from a case report of an incidental diagnosis following an emergency cesarean section, and taking into account the available literature. Emergency interventions and examination of the placenta, even for the smallest IC lesion can ensure timely treatment and improve maternal and fetal outcomes.

## 1. Introduction

Intraplacental choriocarcinoma (IC) is a rare but serious complication of pregnancy. It is generally regarded as the most aggressive trophoblastic disease. Its prevalence is estimated to be around 1/50,000 pregnancies [1]. IC is often diagnosed late and typically occurs after the development of maternal metastasis. The most common recurrent lesions appear to be pulmonary, hepatic, and cerebral. Late diagnosis is thought to be associated with a broad and heterogeneous clinical spectrum. It can range from a complete absence of symptoms to massive vaginal bleeding and fetal or obstetric complications. However, it is associated with an increase in human chorionic gonadotropin (hCG) in the blood and urine. In addition, hCG levels appear to be directly correlated with clinical presentation and survival [2]. Risk factors for IC are maternal age (both <15 years and >45 years old), a history of hydatidiform mole [3], abortion, multiparity, and nulliparity. Massive fetomaternal hemorrhage (FMH) is a serious complication of IC. This event is caused by the break-through of the placental barrier leading to invasion of the myometrial and maternal vascular space by IC tissue [4]. To define FMH, at least 50 mL of fetal blood must enter the maternal circulation [5]. FMH is therefore associated with increased maternal and fetal morbidity and mortality [6]. A possible complication of FMH is fetal anemia. In the past, fetal anemia was usually diagnosed on the basis of a pathognomonic triad: reduced or absent fetal movements, sinusoidal cardiotocographic pattern, and fetal hydrops, with late clinical onset being the main limitation. Currently, it is possible to suspect fetal anemia by assessing the peak systolic velocity of the middle cerebral artery [4]. Other possible complications of IC are retroplacental hematoma, placental abruption, fetal hydrops, fetal growth restriction, or stillbirth [3]. The maternal survival rate for IC is 85–94% without metastases. It drops to 70% with cerebral involvement, 27% with liver metastases, and only 10% if both structures are affected [7]. The aim of this review is to describe the clinical management of this rare condition. In our clinical case, the diagnosis was made after an emergency cesarean section, thanks to the histological examination of the placenta, which showed the presence of a choriocarcinoma through one of the smallest lesions described in the literature. This emphasizes the importance of examining the placenta, even in the absence of evident metastasis, and suggests that even very small lesions can cause significant complications, such as fetomaternal hemorrhage.

## 2. Case Description

**Figure 1:**
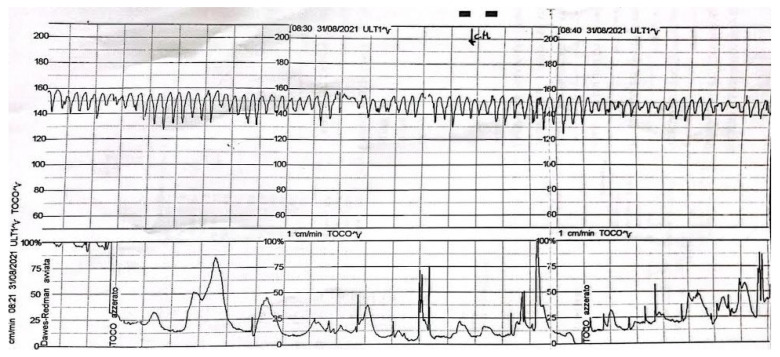
Fetal sinusoidal cardiotocography (CTG) tracing.

A 34-year-old nulliparous woman at 37 weeks’ gestation with a history of fibroid uterus and two previous laparotomic myomectomies came to our attention after a routine examination. On initial evaluation, the pregnancy appeared uneventful, and there were no abnormal values or parameters. Subsequently, during fetal well-being assessment by cardiotocography (CTG), a sinusoidal tracing was evident (Figure 1). The woman was then admitted, and delivery was performed by cesarean section.

**Figure 2:**
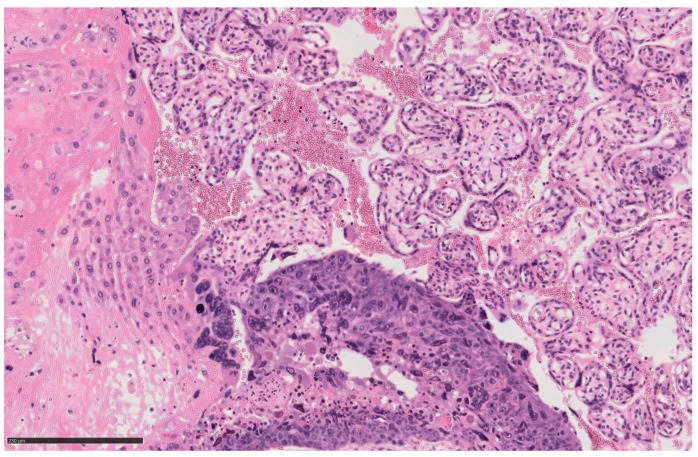
Intraplacental choriocarcinoma histology (hematoxylin and eosin stain). Choriocarcinoma composed of solid sheets of atypical trophoblastic cells with necrosis and surrounded by uninvolved placental chorionic villi can be observed (Microscopic magnification: 17.4× and scale bar-250 µm).

**Figure 3:**
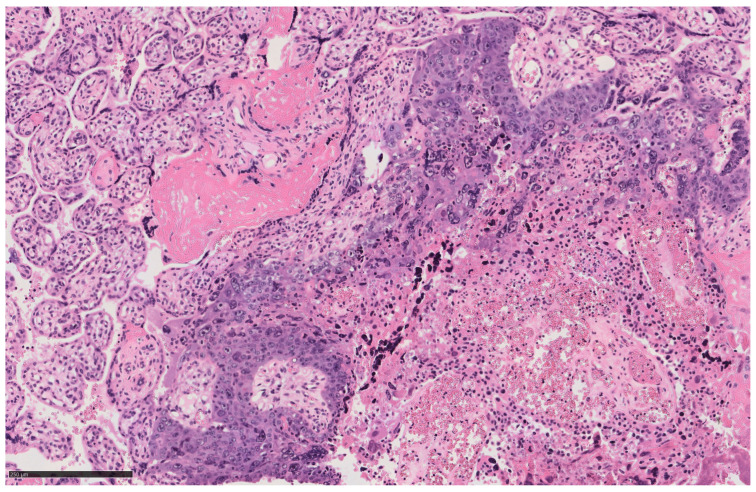
Intraplacental choriocarcinoma. (Microscopic magnification: 14.7× and scale bar-250 µm).

During the surgery, irregular uterine morphology and two bulky fibroids were detected. A live and viable male fetus was delivered by breech. The color of the amniotic fluid was tinged green. Neonatologists assigned an Apgar index of 6 and 7 at 1′ and 5′ minutes and had to resuscitate the baby. The results of cord blood gas analysis showed a hemoglobin (HB) concentration of 4.3 g/dL, a pH of 7.32. and a base excess of −2.9 mmol/L. A blood sample confirmed the infant’s anemia, with a red blood cell (RBC) count of 1.06 × 106/μL, HB: 4.0 g/dL and hematocrit (HC) 12.7%. The neonate was in a state of hypovolemic shock and respiratory failure with pulmonary hypertension, requiring blood transfusion and orotracheal intubation. The placenta weighed 600 g, had a disc diameter of 16 cm, and a marginal insertion of the umbilical cord (with a length of 24 cm). Histological examination of the placenta revealed IC (Figure 2 and Figure 3) with a maximum diameter of approximately 3 mm, which is one of the smallest IC lesions described in the literature.

The parenchyma of the placenta was characterized by aspects of stromal scleroialinosis, perivillous fibrinoid deposits, and areas of recent hemorrhagic leakage. The Kleihauer–Betke test was not performed. To address the concern regarding fetomaternal hemorrhage (FMH) as a potential cause of fetal and newborn anemia, we conducted a thorough exclusion of other potential causes. Specifically, there was no history of maternal trauma, as confirmed through detailed patient interviews and clinical examination. Additionally, placental abruption was excluded, as imaging and clinical assessments showed no signs of placental detachment or related symptoms. Conditions such as preeclampsia and eclampsia were ruled out given the absence of hypertension, proteinuria, or other associated symptoms. Placental abnormalities, including placenta previa and placenta accreta, were also excluded based on imaging findings. Furthermore, tests were conducted to exclude red blood cell membrane disorders and infections that could contribute to anemia, both of which were negative. Based on these signs, the woman underwent a whole-body computed tomography (CT) scan with and without iodinated contrast for cancer staging, and no secondary disease was detected. After one month, a total body CT scan was performed. Repeated monthly maternal and fetal β-hCG quantification was also negative, 5 October 2021: β-hCG < 2.39 mUI/mL (n.v < 10) e AFP 44.6 ng/mL(n.v. < 10), 22 October 2021: β-Hcg < 2.0 mU1/mL (n.v. < 10) e AFP 7.73 ng/mL (n.v. < 10). One year after the delivery, the patient continued her oncological follow-up without treatment. According to the standard approach of high-risk gestational trophoblastic disease follow-up, she underwent a chest, abdomen, and pelvicCT scan which was negative, as well as a breast ultrasound and a β-hCG and AFP dosage. The newborn underwent a magnetic resonance imaging (MRI) scan ten days after birth, which showed no focal areas of altered brain signal, although it was limited by the presence of artifacts due to movement. A cerebral ultrasound was performed the day after birth, which showed only a grade I intraventricular hemorrhage (IVH), typical of premature babies, which was also detected at the next examination after one month. The neuropsychiatric follow-up examination revealed no criticism of posture, reflexes, strength, or movements for both the mother and child.

## 3. Literature Review

### 3.1. Materials and Methods

We selected only papers written in English. All articles that addressed, regardless of study population and methods used, the diagnosis and management of massive FMH caused by IC were considered. Only original papers that reported specific experience data on the topic were included. Studies that described only the physiopathology of the disease and those with inconclusive data were excluded.

### 3.2. Information Sources

Our review was carried out according to the quality standards for narrative reviews, as defined and quantified by “SANRA—a scale for the quality assessment of narrative review articles” [8]. The relevant publications were identified after queries of the following sources: PubMed, Google Scholar, Web of Science, and publishers’ databases, complemented by a cross-check of the reference lists. We used a combination of the search terms “gestational” OR “intraplacental” AND choriocarcinoma” OR “trophoblastic pregnancy disease” AND “fetomaternal haemorrhage” AND “obstetrics outcome” OR “fetal outcome”.

### 3.3. Study Selection

Titles and/or abstracts of studies retrieved using the search strategy were screened independently by two review authors (F.S. and L.N.) to identify studies that met the inclusion criteria. The full texts of potentially eligible articles were retrieved and independently assessed for eligibility by two other review team members (A.E. and F.G.). A manual search of the references of the included studies was conducted to prevent the omission of pertinent research. Any disagreement between them over the eligibility of articles was resolved through discussion with a third (external) collaborator. All authors approved the final selection. No time restriction was applied.

## 4. Results

**Figure 4:**
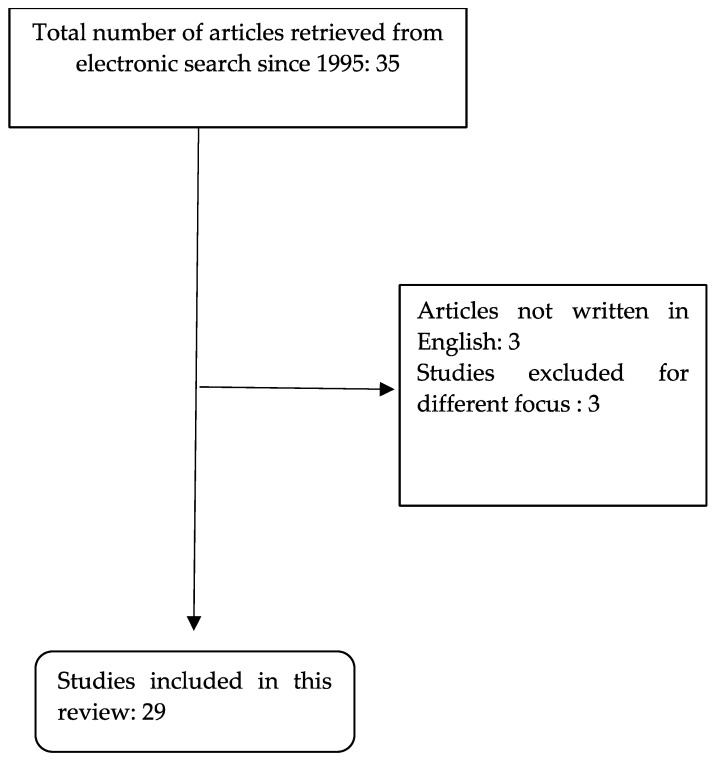
Study selection process.

The literature research yielded 35 articles, from the first published study in 1995 until December 2023 with our described clinical case, with 29 studies included in the final analysis and a total of 29 cases. Study selection for the review is displayed in Figure 4.

The summary Table of included cases is represented in the Supplementary Materials.

The patients’ age range varied from 20 to 47 years (mean age 33.5 years). Most patients were multiparous, some were gravida 2 para 1 (G2P1) or gravida 3 para 2 (G3P2). Obstetric outcomes were highly variable. There were 4 stillbirths (13.3%) and 3 neonatal deaths (10%), corresponding to an overall perinatal mortality of 20.7%. Emergency cesarean section was performed in 40% of cases, and fetal distress or metabolic acidosis occurred in 16.7% of cases. In many cases, fetal anemia (26.7%), reduced fetal movements, (23.3%) or fetal growth restriction (10.0%) were noted. Maternal symptoms, observed in 30% of cases, included persistent vaginal bleeding, fatigue, palpitations, chest pain, and shortness of breath.

The diagnosis of IC was primarily confirmed by histological examination of the placenta. Other tests such as the maternal Kleihauer–Betke test, CT scans, chest X-rays, and maternal serum hCG levels were also useful. The size of the lesions varied, with some as small as <1 cm and others as large as 3 cm (mean size 2.1 cm). Although in many of the reported cases, the exact size of the lesion was not reported on histological examination, our lesion appears to be one of the smallest (approximately 3 mm).

Overall, 43.3% of women received chemotherapy, with EMA-CO (etoposide, methotrexate, actinomycin D/cyclophosphamide, vincristine) being the most commonly used therapy. The proportion of patients with metastases was 43.3%, with remission occurring in 100% of cases after chemotherapy, while in 6.7% of cases (in which chemotherapy had not yet been started or was refused), the mother died. Follow-up after treatment varied in duration (range), with disease-free intervals of several years mentioned in some cases.

## 5. Discussion

This study shows that intraplacental choriocarcinoma (IC) is associated with adverse perinatal outcomes, including a perinatal mortality rate of 23.3%, emergency cesarean sections in 40% of cases, and fetal distress or metabolic acidosis in 16.7%. Maternal or fetal metastases were reported in 43.3% and 3.3% of cases, respectively, with remission achieved in 100% of patients following chemotherapy. The only newborn with metastases died at 38 days of age. IC is frequently associated with fetomaternal hemorrhage (FMH), stillbirth, and maternal/fetal metastases, although the risk factors for these rare complications remain unclear [9,10,11].

Since 1990, only 19 cases of IC associated with FMH have been documented [3,5,12,13,14]. Ryu N. et al. [15] found that IC is more common in women with a history of molar pregnancy [15]. FMH, defined by the amount of fetal blood entering the maternal circulation, can occur as early as the fourth week of pregnancy and may lead to stillbirth, neonatal anemia, or hypoxic-ischemic encephalopathy, though it often remains clinically silent. In our case, despite severe FMH, no secondary metastases were found [16,17,18]. Takahashi et al. [19] reported that IC and FMH coexist in only 37% of cases. Some studies suggest that blood group incompatibility, such as ABO, may help reduce fetal blood loss [20,21]. In our case, the newborn was Rhesus-null positive, and the mother was Rhesus-B positive. The clinical presentation of FMH is broad, with non-specific symptoms. Sinusoidal patterns associated with IC are well documented, as in the case of Hsiu-Huei Peng et al. [22], where stage 3 IC was diagnosed by CT scan, and the newborn required intubation and transfusion.

Stillbirth, a potential sign of IC, was reported by Monteiro et al. [23] and Chui M. Lam et al. [24], where severe anemia and fetomaternal transfusion (106 mL) were noted. These cases highlight the serious fetal complications associated with IC, such as fetal hydrops, scalp edema, and ascites.

Significant reports from the literature [3] describe another common IC complication: severe vaginal bleeding after labor. Pulmonary metastases recurred, emphasizing their frequency in IC cases. A peculiarity of our case is the absence of secondary metastases despite the picture of FMH, as in 86.7% of cases, metastases are found at the time of diagnosis [24]. The case described by Thagard Andrew S. et al. [25], for example, describes a 21-year-old woman who suffered from chest pain and dyspnea and in whom more than 40 pulmonary opacities, each 2–4 cm in size, were found on X-ray. It was only after a cesarean section and histological examination of the placenta that IC was diagnosed [25]. On the other hand, a case similar to ours was described by Henningsen et al. [1], in which the discovery of a pathological CTG led to an emergency cesarean section. The newborn’s blood test showed severe anemia. After receiving the IC diagnosis, both the mother and the newborn underwent a radiological examination, which was negative for metastases [1]. In the work presented by Yuji Koike et al., no metastases were found and only β-hCG levels were checked [21]. In the absence of specific symptoms and/or secondary disease, a reliable diagnosis was possible thanks to histopathological examination of the placenta. This is important to emphasize as in about half of IC cases, the placenta is not examined and thus the possibility of early diagnosis is lost [24]. Cancer screening limited to a macroscopic examination of the placenta is inadequate and difficult as the lesion may be small and therefore more likely to appear as a benign lesion or striated area [9,26,27].Another illustrative example of the importance of histological examination of the placenta in determining the diagnosis of IC is the case presented by Mosayebi Z. et al., in which both the child and the mother had choriocarcinoma [28], or the case presented by Takai N. et al. [29], in which the presence of fetal anemia led to the diagnosis of FMH. To clarify the main cause, a histological examination of the placenta was performed, which again revealed IC [29]. Gestational choriocarcinoma is a trophoblastic malignant neoplasm that is composed of neoplastic syncytiotrophoblast, cytotrophoblast, and intermediate trophoblast, and usually occurs in the uterus, but also occurs at ectopic or metastatic sites [30]. Grossly, the neoplasm appears as a single or multiple dark red, solid, friable masses with areas of hemorrhage; intraplacental choriocarcinoma can be a subtle lesion and is often mistaken for a placental infarct or thrombus. Microscopically, it shows solid aggregates of atypical syncytiotrophoblast, cytotrophoblast, and intermediate trophoblast in the absence of chorionic villi; in intraplacental choriocarcinoma, the tumor is surrounded by non-neoplastic villi. Hemorrhage, necrosis, and lymphatic infiltration often occur [31,32]. Neoplastic syncytiotrophoblast and intermediate trophoblast display strong immunoreactivity for hCG, hPL, inhibin, MCAM, SALL4, HLA-G, MUC4, and p63; the Ki-67 proliferation index is typically high (>90%) [32]. The differential diagnosis includes placental site trophoblastic tumor, an epithelioid trophoblastic tumor, a non-gestational choriocarcinoma of germ cell origin, a somatic carcinoma with trophoblastic differentiation, a complete mole, an exaggerated placental site, and placental site nodule/plaque [32]. It is important to emphasize the role of β-hCG dosage in the therapeutic management of a woman with IC. A significant review by Jiao et al. suggests that an increase in β-hCG levels above the 95th percentile during pregnancy may indicate the presence of gestational trophoblastic neoplasia (GTN). However, it appears to be easier to understand and diagnose the consequences of such conditions if β-hCG levels are monitored during labor [2]. As early as 1992, Duleba et al. [33] noted that, with strict monthly β-hCG monitoring in both the mother and the newborn, chemotherapy may not be necessary in cases without metastases or elevated β-hCG levels, as seen in our case [26,33,34,35]. Both the mother and the newborn in our case had normal β-hCG levels one year after delivery. In contrast, a clinical classification system is used for patients with metastases [26]. Treatment of IC typically involves chemotherapy, often combined with surgery and/or radiotherapy. Low-grade cases can be treated with a single agent such as methotrexate, while high-grade forms require polychemotherapy [33].

## 6. Conclusions

In conclusion, intraplacental choriocarcinoma is a rare but potentially life-threatening condition that requires timely diagnosis and management to improve maternal and fetal outcomes. Our case highlights the importance of histological examination of the placenta, even in the absence of obvious clinical signs. Moreover, the absence of metastasis in both the mother and the newborn, despite the massive hemorrhage, is noteworthy and may contribute to further discussions on the heterogeneous progression of intraplacental choriocarcinoma. Early detection and close follow-up, including β-hCG monitoring, are crucial for preventing complications. Multidisciplinary collaboration between obstetricians, pathologists, and oncologists plays a pivotal role in ensuring the best possible prognosis for both mother and child.

## Data Availability

The original contributions presented in the study are included in the article. Further inquiries can be directed to the corresponding author.

## Supplementary Materials

The following supporting information can be downloaded at: https://www.mdpi.com/article/10.3390/diagnostics14212454/s1, Table S1: Summary and characteristics of included cases. References [1,2,3,4,5,7,9,10,11,12,19,20,21,22,23,24,25,26,27,29,33] are cited in the Supplementary Materials.
