# Supplementary material for: Massive Fetomaternal Hemorrhage Caused by an Intraplacental Choriocarcinoma: Case Report and Review of the Literature
Source: Diagnostics (Basel). 2024 Nov 1;14(21):2454. doi: 10.3390/diagnostics14212454 (PMC11544788; doi:10.3390/diagnostics14212454)
Supplement: Supplementary file 1 [file diagnostics-14-02454-s001.zip › diagnostics-3244939-supplementary.pdf]

Supplementary Table S1: Summary and characteristics of included cases

AUTHORS	AGE	HISTOLOGICAL EXAMINATION	SIZE OF LESION	PARITY	DIAGNOSIS METHODS	TREATMENT AND OUTCOME IN MOTHER	GA	OBSTETRICAL OUTCOMES	FETAL/MATERNAL SYMPTOMS	FETAL METASTASIS
Anna-Karina Aaris Henningsen et al. [1]	47	PlacentaHistological examination : IC	3cm	P7	Histological examination	No metastasis, follow-up	35 + 6 weeks	Emergency cesareansection	Fatigue and palpitations Decreased fetal movements, patological CTG, fetal anemia and sepsis	infant remained hospitalized for 1 month. The serum levels of both alfa-foetopr

										otein and hCG were at all times normal why no ultrasound, X-ray or CT scans were performed in search for metastases. The infant showed no signs of neurological damage ..
L. Jiao et al. [2]	32	PlacentaHistological examination: focal area of IC	n.r.	G5P4	positive maternal Kleihauer-Betke test, histological examination	No metastasis, follow-up . The mother was	40 weeks	delivered a stillborn infant	Uncomplicatedgestation	No evidence

						disease-free for nine years and had two subsequent uncomplicated pregnancies				
L. Jiao et al. [2]	39	PlacentaHistological examination: IC within this lesion	n.r.	G2P1	Histological examinations	No metastasis, follow-up. Disease-free for 8.5 years	38 weeks	delivered a stillborn infant	Uncomplicatedgestation	No evidence
L. Jiao et al.[2]	37	PlacentaHistological examination:1st) focus of IC 2nd) IC	1st)n.r. 2nd) n.r.	G2P1	1st) Histological examinations 2nd) Histological examinations	1st) No metastasis, follow-up. 2nd) No metastasis, follow-up.	1st) 35 weeks 2nd)Inducedlabour at 38 weeks	1st) delivered a stillborn infant 2nd) induced labour at 38 weeks	Uncomplicatedgestation	No evidence
L. Jiao et al.[2]	32	PlacentaHistological examination : IC	<1cm	G1P0	Histological examinations	No metastasis, follow-up.	40 weeks	Emergency cesareansection	Infantseverelyanemic	No evidence

Mariana Simões et al. [3]	35	uterine curettagebiopsyconfirmedchoriocarcinoma	n.r.	gravida 2 para	Histological examinations. Abdominal and thoracic computerized tomography	pulmonary metastasis, remission after chemotherapy	39 weeks	Vaginal birth, mother presented to the obstetric emergency department with persistent vaginal bleeding since delivery	Uncomplicated gestation. Infant was severely anemic, grunting, with severe pallor, cold extremities, hypoactivity and acidosis	No evidence
KaaiAso et al. [4]	27	PlacentaHistological examination: IC	n.r.	G2P1	-CTG with sinusoidal rate pattern<br>- MCA-PSV increased<br>- HbF cells in the maternal circulation was 8.9%, FMH<br>- hCGlevels	No metastasis, seven cycles of chemotherapy	37 weeks	emergency cesarean section, he died of hypovolemic shock 13 h after birth	in fetalmovement and	An autopsy of the neonate showed normal fetal structure and no metastases.
QinShe et al. [5]	21	PlacentaHistological examination: IC	n.r.	G1P0	CTG sine wave,positive maternal Kleihauer-Betke test, histological examination	patient refused CT scans ,chest X-ray, and therapy, died at 109 days	35 weeks	Emergencycesareansection	Reducedfetalmovements	From a neonatal perspective, 6 days after birth, the

						postdelivery with number of metastases in the lung, uterus and cerebral hemorrage				infant's b-HCG level was 30.4 IU/L (normal range to normal by day 41. At the time of writing, the baby remains free from disease
Blanca Moreno-Gomez et al.[7]	22	PlacentaHistological examination : IC	n.r.		chest X-ray, CT scans, histological examinations	Lung metastases, Remission after twelve cycles of chemotherapy (EMA-CO)	34 weeks	Plannedcesareansection	Spontaneous metrorrhagia and hemoptysis, with dyspnea	No evidence

B Hookins and A Vatsayan [9]	31	PlacentaHistological examination : IC	11mm	G1P0	CTG sinusoidal pattern β-hCGlevels 3 weeks after cesarean section, istological examinations, CT scans	large soft-tissue mass distending the uterine cavity, with possible extension into the right adnexa, remission after chemotherapy (EMA-CO)	38 weeks	emergency cesarean section, Bakri balloon for HPP.<br>Three weeks post-partum persistent vaginal bleeding	reduced fetal movements. The neonate was pale and had significant anemia	the child is healthy with no complications
Kurt Benirschkeet al.[10]	21	PlacentaHistological examination : IC	2cm	G4P3	chest X-ray, β-hCGlevels 20 days after cesarean section	Pulmonary metastases, remission after combination chemotherapy (VP-16, actinomycin, and methotrexate	37 weeks	cesarean section, She presented 20 days later complaining of vaginal bleeding	Uncomplicatedgestation	The infant is healthy and his serum beta hCG has been consistently undetectable.

						followed by vincristine and cytoxan)				
FM Wilcock and RA Kadir [11]	28		n.r.	G1P0	positive maternal Kleihauer-Betke test, fetal autopsy		38 weeks and 1 day	emergency cesarean section, heartbeat was obtained only after 25 minutes following blood transfusion. The infant died 5 hours after birth due to severe metabolic acidosis	reduced fetal movements, fetal bradycardia	No evidence
Andreitchouk, Alexandre Evgenievich et al. [12]	36	Autopsy (newborn) : 2 huge tumors (7 x 6 x 6 and 10 x 6 x 7 cm) in the liver, multiple lung metastases, and 400 ml of blood-tinged ascites	Not done	G3P1	ultrasonographic examination, autopsy, transvaginal ultrasonography, chest X-ray	Liver metastasis with a maximum size of 7 X 6 X 6 cm. Hypoechoic tumor in the myometri	At term	cesarean section due to cephalopelvic disproportion	history of a hydatidiform mole	At 12 days of age, hepatomegaly was noticed, and an ultrasonographi

						um and a lutein cyst in her left ovary (4 x 4 cm).Lung metastases, up to 1 .0 cm in size. Her laboratory data revealed severe anemia and remarkably high levels of hCG. Complete remission after fourteen courses of chemotherapy (EMA-CO)				c examination revealed 2 hyperechoic tumor lesions with an irregular border in her liver. At 20 days of age, brain and lung metastases were also detected. newborn died at the age of 38 days.

Takahashi, Hironori et al. [19]		PlacentaHistological examination : IC	n.r.			No metastasis, follow-up.	38 weeks	Emergencycesareansection	reduced fetal movements, abnormal fetal heart rate patterns. The infant was severely anemic	No evidence
Nino Solomonia et al. [20]	21		n.r.	G1P0	Kleihauer-Betke test		28 weeks	Emergencycesareansection	reduced fetal movements, 3 days before delivery the mother had general malaise, illness and abrupt development of generalized edema. The infant was severely anemic, with severe metabolic acidosis	No evidence

YujiKoike et al. [21]	31	Histology of the placenta: IC	n.r.	G0P0	Pathological CTG, histologicalexaminations	No metastasis, follow-up	39 weeks	Emergencycesarean section	fetal anemia	The infant's chest and abdominal x-ray, brain magnetic resonance imaging study, and ultrasonography findings revealed no metastases
Peng, Hsiu-Huei et al. [22]	34	uterine curettagebiopsyconfirmedchoriocarcinoma	n.r.	G3P2	CTG with sinusoidal rate pattern Histological examinations CT scans	heterogenous mass in the uterine cavity and multiple lung metastases.Complete remission after eight	37 weeks	emergency cesarean section. The mother returned 2 weeks postpartum because of profuse vaginal bleeding	reduced fetal movements, neonate was pale with marked anemia	No evidence

						cycles of chemotherapy (EMA-CO)				
Monteiro, S et al. [23]	33	Histology of the placenta: IC	21x19mm	G2P0	Kleihauer-Betke test positive, histological examinations, fetal autopsy	No metastasis, follow-up. She had a subsequent successful term delivery	36 weeks and 6 days	induced labour at 36.6 weeks with a normal vaginal birth of a stillborn infant, post-partum haemorrhage from uterine atony	Reducedfetalmovements	No evidence
Lam, Chui M et al. [24]	30	Histology of the placenta: IC uterine curettage biopsy confirmed choriocarcinoma	1cm	G2P1	Kleihauer–Betke test positive, histological examinations, fetal autopsy	No metastasis, two courses of chemotherapy (methotrexate)	35+ weeks	induced labor at 36.6 weeks with a normal vaginal birth of a stillborn infant due to massivefeto-maternal hemorrhage. The mother presented 7 weeks after her delivery with heavy bleeding	abdominal distension and also complained of subcostal discomfort, fetal death in uterus	No evidence
Lam, Chui M et al. [24]	27	Notperformed	Not done	G1P0	Kleihauer–Betke test positive, β-hCGlevel, Chest x-ray	pulmonary metastases, remission after three cycles of	39 weeks	Vaginal birth	The baby was anemic and suffered from heart failure. The mother returned 3	No evidence

						chemotherapy			weeks after delivery with pleuritic chest pain and shortness of breath	
Thagard, Andrew S et al. [25]	21	Histology of the placenta: IC	3,1 cm	gravida 1, para 0	chest x-ray, β-hCGlevel, MCA-PSV increased, Kleihauer–Betke test positive, histological examinations	pulmonary metastases, remission after five cycles of chemotherapy (3 CHAMOCA, 2 MBE)	36 weeks	Plannedcesareansection	significant chest pain and shortness of breath, fetal anemia	No evidence
H T Nagel et al. [26]	30	Histology of the placenta: areas of infarctionand intraparenchymatous hemorrhage	n.r.	G2P0	Kleihauer–Betke test positive. AFTER ABORTION β-hCGlevel, Ultrasound and magnetic resonance imaging	lesion in the uterine cavity, no metastases, remission after six cycles of chemotherapy (EMA-CO)	40 weeks	Induction of labor;She gave birth to a stillborn child. Three months after giving birth, she had an abortion. Three years later she gave birth to a healthy child.	intrauterine fetal death at the time of birth, history of a complete hydatidiform mole	No evidence

Suzanne M. Jacques et al. [27]	20	Histology of the placenta: IC	n.r	G3P3	Histological examinations	No metastasis, Methotrexate	38 weeks	Vaginalbirth		No evidence
Suzanne M. Jacques et al.[27]	34	Histology of the placenta: IC	n.r.	G4P3	Histological examinations	No metastasis, Methotrexate	40 weeks	Vaginalbirth		No evidence
Suzanne M. Jacques et al.[27]	24	Histology of the placenta: IC	n.r.	G7P4	Histological examinations	No metastasis, follow-up	40 weeks	Cesareansection		No evidence
Suzanne M. Jacques et al.[27]	22	Histology of the placenta: IC	n.r.	G1P1	β-hCGlevel, histological examinations	Pulmonary metastases, chemioterapy (EMA-CO)	35 weeks	Vaginalbirth	fetal anemia	No evidence
Suzanne M. Jacques et al. [27]	29	Histology of the placenta: IC	n.r.	G2P1	β-hCGlevel, histological examinations	Pulmonary metastases, chemioterapy (EMA-CO)	25 weeks	Cesareansection		No evidence

Takai, N et al. [29]	35	Histology of the placenta: IC	n.r.	G1P0	Histological examinations	No metastasis, follow-up	37 weeks	Emergencycesareansection	IUGR, fetal distress, fetal anemia	newborn was treated subsequently with blood transfusions and responded well. He had no evidence of choriocarcinoma metastases following serum b-hCG concentration and CT-scan investigations
A J Dulebaet al.[33]	30	Histology of the placenta: IC	n.r.	P0G0	Histological examinations	No metastasis, follow-up	36 weeks	cesarean section, 18 months after the mother was pregnant again	IUGR, fetal distress	No evidence

MBE (methotrexate, bleomycin and etoposide) CHAMOCA (cyclophosphamide, hydroxyurea, actinomycin-D, methotrexate, vincristine and doxorubicin) EMA/CO (etoposide, methotrexate, actinomycin D/cyclophosphamide, vincristine), n.r. (not reported).
